# Fully Integrated Silicon Photonic Erbium-Doped Nanodiode for Few Photon Emission at Telecom Wavelengths

**DOI:** 10.3390/ma16062344

**Published:** 2023-03-15

**Authors:** Giulio Tavani, Chiara Barri, Erfan Mafakheri, Giorgia Franzò, Michele Celebrano, Michele Castriotta, Matteo Di Giancamillo, Giorgio Ferrari, Francesco Picciariello, Giulio Foletto, Costantino Agnesi, Giuseppe Vallone, Paolo Villoresi, Vito Sorianello, Davide Rotta, Marco Finazzi, Monica Bollani, Enrico Prati

**Affiliations:** 1 L-NESS, Department of Physics, Politecnico di Milano, Via Francesco Anzani 42, I-22100 Como, Italy; 2 Istituto di Fotonica e Nanotecnologie, Consiglio Nazionale delle Ricerche, Piazza Leonardo da Vinci 32, I-20133 Milan, Italy; 3 Consiglio Nazionale delle Ricerche, Istituto per la Microelettronica e i Microsistemi (CNR-IMM), Via Santa Sofia 64, I-95123 Catania, Italy; 4 Department of Physics, Politecnico di Milano, Piazza Leonardo da Vinci 32, I-20133 Milan, Italy; 5 Department of Electronics, Information and Bioengineering, Politecnico di Milano, Piazza Leonardo da Vinci 32, I-20133 Milan, Italy; 6 Department of Information Engineering, Università degli Studi di Padova, Via Gradenigo 6B, I-35131 Padua, Italy; 7 Photonic Networks and Technologies Lab., Consorzio Nazionale Interuniversitario per le Telecomunicazioni (CNIT), I-56124 Pisa, Italy; 8 CamGraPhIC Srl, Via G. Moruzzi 1, I-56124 Pisa, Italy; 9 TeCIP Institute, Scuola Superiore Sant’Anna, Via G. Moruzzi 1, I-56124 Pisa, Italy; 10 Department of Physics “Aldo Pontremoli”, Università degli Studi di Milano, Via Celoria 16, I-20133 Milan, Italy

**Keywords:** silicon photonics, quantum key distribution, SOI diode, erbium doping

## Abstract

Recent advancements in quantum key distribution (QKD) protocols opened the chance to exploit nonlaser sources for their implementation. A possible solution might consist in erbium-doped light emitting diodes (LEDs), which are able to produce photons in the third communication window, with a wavelength around 1550 nm. Here, we present silicon LEDs based on the electroluminescence of Er:O complexes in Si. Such sources are fabricated with a fully-compatible CMOS process on a 220 nm-thick silicon-on-insulator (SOI) wafer, the common standard in silicon photonics. The implantation depth is tuned to match the center of the silicon layer. The erbium and oxygen co-doping ratio is tuned to optimize the electroluminescence signal. We fabricate a batch of Er:O diodes with surface areas ranging from 1 µm × 1 µm to 50 µm × 50 µm emitting 1550 nm photons at room temperature. We demonstrate emission rates around 5 × 10${}^{6}$ photons/s for a 1 µm × 1 µm device at room temperature using superconducting nanowire detectors cooled at 0.8 K. The demonstration of Er:O diodes integrated in the 220 nm SOI platform paves the way towards the creation of integrated silicon photon sources suitable for arbitrary-statistic-tolerant QKD protocols.

## 1. Introduction

Quantum key distribution (QKD) is one of the best-known quantum communication protocols. Since its first theoretical implementation based on single photon sources, many progresses have been made [1]. Among these, one of the most important consisted in the introduction of the decoy state protocol, which enabled the use of lasers in the experimental implementations of QKD protocols [2]. Unfortunately, even if silicon photonic has been catching on in recent years, a full silicon implementation is still lacking since off-chip laser sources are still required, and only integrated passive components and modulators are currently available [3,4]. In principle, III-V lasers could be integrated on a silicon chip through wafer-bonding techniques [5] or by hybrid integration through flip-chip bonding [6]. This at the price of increasing the fabrication cost and the complexity of the process. A possible solution is suggested by a recent work [7] that demonstrates decoy state QKD using arbitrary photon statistics. Such an option opens the way to the employment of non-laser sources in silicon chips, such as the SOI LED photon source based on Er-O complexes presented in this work. Such a source is supposed to have a binomial photon-number distribution and, in a QKD experiment, it should work in pulsed mode with an average number of photons per pulse less than one. Erbium-based diodes in bulk silicon have been extensively explored for high power applications in recent decades [8,9,10]. Regarding SOI platforms, a photoluminescence (PL) characterization of erbium-related defects has been performed on a 2 µm silicon-on-insulator [11] platform but erbium-doped diodes on SOI have not yet been reported in the scientific literature. The shift from bulk silicon to the 220 nm SOI platform is required in sight of integrating this source in a silicon photonic chip [12]. In fact, Si photonics provides a comprehensive toolkit of photonic building blocks including modulators, phase shifters and fiber couplers to design complex circuits. This enables advanced photonic devices featuring high performance and functionality for a Size, Weight and Power (SWaP) reduction which is fundamental for space applications. Moreover, consolidated CMOS technologies can provide improved yield and scalability [13]. Previously, we have characterized the room-temperature PL of Er-O complexes at extremely low concentration in bulk silicon [14], explored the Er diffusion by atom probe tomography [15] and designed Er-based resonant cavities [16] within the framework of employing Er-related defects in silicon for single photon applications [17]. Here, we report about the fabrication and electroluminescence characterization at room temperature of erbium diodes on a 220 nm silicon-on-insulator. We have opted for a completely CMOS compatible process to match the semiconductor industry requirements. FDTD simulations have been carried out to estimate the total number of photons generated in the nanodiode and to show the tolerance of such devices to a slight discrepancy between the fabricated features and the nominal parameters. The characterization of the electroluminescence yield is carried out at room temperature by using a superconducting SPAD.

In our devices, erbium is implanted at 300 keV to achieve a peak concentration in the center of the Si layer. We confirm that the strongest enhancement of the electroluminescence signal is obtained with an O:Er ratio equal to 9 [18]. We find an emission rate of 5 $\times 10^{6}$ photons/s for a 1 µm × 1 µm device in open space. According to our results, the implantation of Er in SOI represents an important first step towards the integration of 1550 nm-weak sources in silicon photonics.

This work is divided into three Sections. In Section 2, we describe the fabrication of the devices, paying special attention to the erbium and oxygen co-doping, both critical with respect to the electroluminescence signal. In Section 3, we will provide electromagnetic simulations to evaluate the vertical emission of the devices. A parametric study is also presented to estimate the impact of a fabrication error with respect to the nominal device parameters. In Section 4, we will report the experimental results. The last Section summarizes the conclusions.

## 2. Materials and Methods

### 2.1. Nanofabrication Process

The device consists of a silicon planar p-n junction with a central region, nearby the depletion area, co-doped with erbium and oxygen atoms. Luminescent erbium-doped silicon has been studied in the literature since erbium ions in their 3+ state emit photons at 1540 nm wavelength [9]. This emission is due to the 4*f* inter shell radiative transition from the first excited state ${}^{4}I_{13/2}$ to the ground state level ${}^{4}I_{15/2}$. Such transition is forbidden by parity selection rules but, when Er${}^{3+}$ ions are inserted in Si, the interaction with the crystal field and the lowered symmetry produce a mixing of states with different parities and the transition becomes allowed [18].

**Figure 1:**
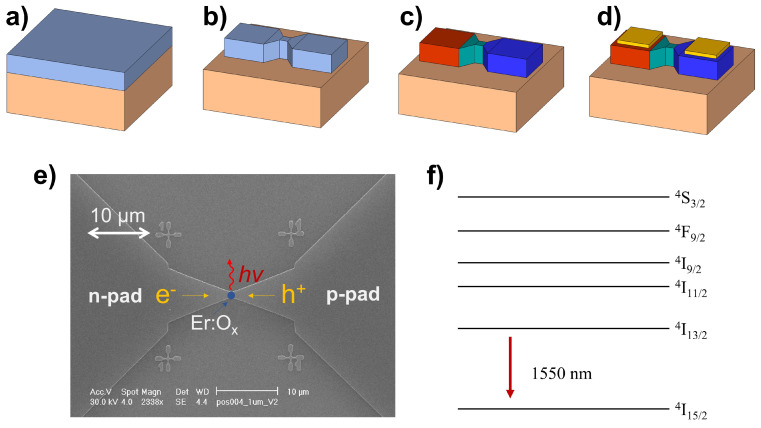
(**a**) Silicon on insulator wafer, the silicon layer is 220 nm-thick and it lies on a 3 µm-thick SiO${}_{2}$ buried layer. (**b**) Mesa of the device defined by electron beam lithography (30 keV) using a positive resist (PMMA). The etching is performed via a Bosch process. (**c**) Sketch illustrating the doping of the device. In red, the p-area doped with boron, in blue, the one doped with phosphorous, and, in green, the region co-doped with erbium and oxygen. (**d**) Final device. In yellow, the electrical contacts made of titanium and gold. (**e**) Scanning electron microscopy image of the device. Our simulations indicate that most of the photons are confined in the silicon layer and just 6% of them are emitted in open space in the vertical direction. (**f**) Energy level structure in Er${}^{3+}$, the transition exploited in our device is displayed.

When a potential difference is set between the p and n regions, electron-hole pairs are injected in the device. They eventually recombine close to the depletion zone, possibly causing the promotion of the erbium ions into the excited state. The mechanism underlying this process consists in an Auger recombination process of a hole in the valence band and an electron that is trapped in a localized level in the silicon band gap. This level is introduced by the Er${}^{3+}$ doping. The energy of this trap-level is at 0.15 eV below the conduction band [19]. The electron-hole recombination leads to an energy transfer to the Er ions [20]. Finally, the radiative de-excitation from the ${}^{4}I_{13/2}$ to the ${}^{4}I_{15/2}$ level of Er${}^{3+}$ is responsible for photon emission (Figure 1f).

Unfortunately, it is well-known that the luminescence from the Er${}^{3+}$ ions is suppressed at room temperature since non-radiative losses dominate the de-excitation process. To circumvent this issue, oxygen co-doping is exploited to form Er-O complexes.

The main consequence is the enhancement of the Er luminescence yield at cryogenic as well as at room temperature [10].

Other kinds of co-doping such as F and N have been studied in the literature. These dopants have a similar effect as the oxygen co-doping on the luminescence at cryogenic temperature, but the oxygen doping offers a smaller temperature quenching compared to other dopants [21]. Concerning the fabrication, the devices are made through a top-down process performed by a combination of electron beam lithography, reactive ion etching, e-beam deposition and ion implantation as displayed in Figure 1. It is worth noting that all the mentioned fabrication steps are CMOS compatible, thus feasible in a standard silicon foundry.

The entire process is performed on a 220 nm-thick Si layer on a 3 µm-thick SiO${}_{2}$ buried oxide layer (Figure 1a). The first step is the definition of the mesa structure by electron beam lithography and reactive ion etching (Figure 1b) [22,23]. Next, dopants are introduced by ion implantation and activated through a rapid thermal annealing process (Figure 1c). Electron beam lithography exposures and SiO${}_{2}$ hard mask evaporations are used to open windows only in the areas where dopants need to be implanted. To get rid of the hard mask, an HF bath is performed. The n-area is doped with phosphorous, whereas the p-area with boron. The optically active area is doped with erbium and oxygen. Further details about the doping process and specifics are described in the next section. The final step consists in the definition of the two electrical contacts on top of the n- and p- doped regions. To pattern the electrodes, we used a standard lift-off process. A positive resist is spin-coated on top of the sample. The electrodes are then exposed using an electron beam lithography system. An e-beam evaporator is used to deposit 5 nm of titanium and 150 nm of gold. Finally, a lift-off is performed to remove the resist. The titanium layer is introduced before the gold contact to improve the gold adhesion on silicon (Figure 1d) [24].

A batch of samples with three different sizes of the erbium-doped area is fabricated and characterized. The doped areas correspond to 1 µm × 1 µm, 15 µm × 15 µm, 50 µm × 50 µm squares, respectively.

### 2.2. Doping Implantation

The 220 nm SOI samples are implanted at room temperature by using a 400 kV High Voltage Engineering ion implanter. While in the case of B, P and Er implants a solid source is used, O implants are made adopting a gaseous source. The p-type region of the device is obtained by a 30 keV B implantation with a dose of 4 × $10^{14}$ cm${}^{-2}$, whereas the n-type region is defined by a 70 keV P implantation with a dose of 3.7 × $10^{14}$ cm${}^{-2}$. After the implantation, the dopants are electrically activated through a 1 h annealing at 920 °C. Er ions are then implanted at 300 keV in order to localize the Er concentration peak at the center of the 220 nm Si layer. The Er dose is set at 1 × $10^{13}$ cm${}^{-2}$, resulting in an Er concentration peak of 1 × $10^{18}$ cm${}^{-3}$, below the Er solid solubility in crystalline Si [25]. O ions are finally implanted at an energy of 40 keV in the Er doped region to overlap the two doping concentration profiles. Previous studies have demonstrated that the maximum luminescence enhancement is achieved with O:Er ratios in the 1–10 range [26,27,28]. Accordingly, the O dose is varied in the range 0.35–1.4 × $10^{14}$ cm${}^{-2}$. After the implantation, all samples are annealed in a N${}_{2}$ atmosphere environment for 30 min to remove the implantation damage and optically activate the erbium ions. We investigated two different annealing temperatures, 900 °C and 920 °C, to determine the one that provides the highest Er:O electroluminescence yield.

## 3. Electromagnetic Modelling

An electromagnetic modelling is carried out to evaluate the vertical photon emission in open space and to estimate the device tolerance with respect to a deviation from the nominal parameters. The simulations are performed by the software Ansys Lumerical FDTD 2022 R1.2 [29].

**Figure 2:**
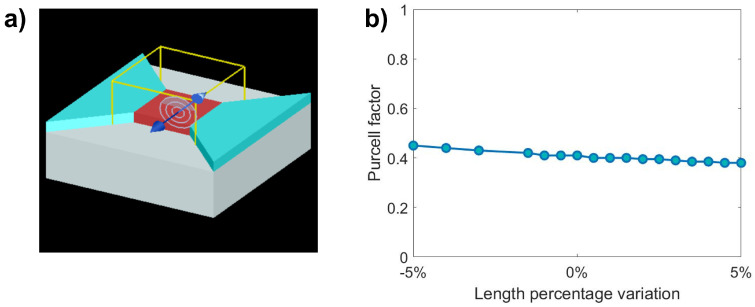
(**a**) Layout of the device simulated with FDTD. In red, the optically active part of the device is represented. This is simulated as a 1 µm × 1 µm undoped silicon square with dipole sources in it. The silicon oxide layer is the grey box and the contacts (in blue) are modelled as doped silicon regions. The blue arrow indicates the orientation of the dipole source whereas the grey concentric lines are the dipole radiation pattern. (**b**) Modification of the Purcell factor considering a variation of the lateral size of the optically active area.

The simulated design is shown in Figure 2a. The active area consists in undoped Si ($n_{\mathrm{Si}}$ = 3.48 at 1550 nm) with a thickness equal to 220 nm. The device is placed on top of a 2 µm thick silicon oxide layer ($n_{\mathrm{SiO}2}$ = 1.44 at 1550 nm) to reproduce the SOI platform.

The presence of erbium ions in silicon is simulated by a modification of the real part of the Si refractive index. The light source is modelled as an oscillating dipole in the silicon layer. We evaluated the fraction of power emitted vertically with respect to the total emission. The adopted boundary conditions were perfectly matched layer (PML) boundary conditions. Furthermore, the Purcell factor is calculated to check that the shape of the device is neither suppressing nor enhancing the emitted power significantly. A Purcell factor less than one indicates that the environment is suppressing the spontaneous emission with respect to the free space case, while a value grater than one corresponds to an increment.

We have found that the percentage of radiation emitted upward remains stable around a value of 6%, regardless of the sample size. The two smaller geometries, which have a Purcell factor of 0.41 and 0.72, respectively, do not promote any increase of spontaneous dipole emission, as they are not resonant with the emitted wavelength. The largest has a Purcell factor of 1.16, still far from optimal resonant conditions.

A parametric study is conducted for the smallest of the fabricated structures, to check the tolerance in the case the fabrication deviates from the nominal size. For the smaller sample, the length of the cuboid is modified, considering a nominal length of 1000 nm and assuming a possible error up to ±5%. The evaluated range is therefore from 950 nm to 1050 nm, with a step size of 5 nm. As shown in Figure 2b, the Purcell Factor varies smoothly from 0.46 to 0.38 with no resonances. This suggests that in case of possible small size deviations, the diode will continue to behave according to expectations.

## 4. Experimental Results

We will now discuss the experimental results based on the fabricated devices. First, we will provide the experimental characterization of the effectiveness of the oxygen doping. Next, we will move to the electroluminescence observed in the devices.

### 4.1. Characterization of Oxygen Doping

In order to understand the best oxygen co-doping dose, three non-fabricated 220 nm SOI samples are doped with erbium and oxygen and then characterized by PL spectroscopy. The erbium doping and oxygen implantation are performed in the same conditions applied to the devices described in the previous section, namely using an energy equal to 300 keV and a dose of 1 × $10^{13}$ Er cm${}^{-2}$ for erbium, whereas the oxygen is implanted with an energy of 40 keV and a dose range between 0.35–1.4 × $10^{14}$ O cm${}^{-2}$ to understand the conditions leading to the best emission performance.

**Figure 3:**
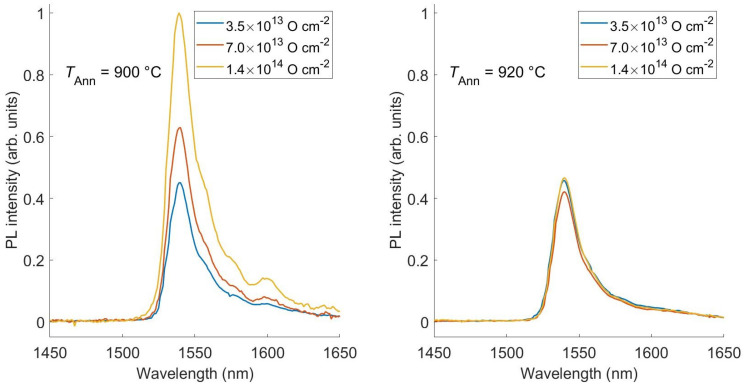
Photoluminescence spectra recorded for three different oxygen co-doping doses. On the left, samples are annealed for 30 min at a temperature *T*${}_{\mathrm{Ann}}$ = 900 °C. On the right, the annealing is performed at 920 °C for the same amount of time. All the signals are normalized with respect to the PL maximum obtained with a dose equals to 1.4 × $10^{14}$ O cm${}^{-2}$ and the annealing temperature equals to 900 °C.

PL measurements are carried out by pumping with the 325 nm line of an HeCd laser. The laser beam is chopped at a frequency of 11 Hz through an acousto-optic modulator. Then, the luminescence signal is focused on the aperture of a single grating monochromator and detected by a liquid-nitrogen-cooled Ge detector. Finally, the signal coming from the detector is measured by a lock-in amplifier using the acousto-optic frequency as a reference in order to improve the signal-to-noise ratio. Low temperature measurements are performed by using a closed-cycle He cryostat with the samples kept under vacuum. In Figure 3, the PL spectra are shown for samples having different O contents and annealed at 900 °C (Figure 3 left panel) and 920 °C (Figure 3 right panel). Thesemeasurements are performed at 11 K.

The PL spectra of the samples annealed at 900 °C (Figure 3 left panel) show the typical features of the Er emission, with a main peak at 1540 nm and a few minor peaks corresponding to the different optical transitions from the ${}^{4}I_{13/2}$ to the ${}^{4}I_{15/2}$ Er multiplets. The PL intensity increases with the O content, confirming that an O-rich environment enhances the Er luminescence yield. The maximum PL yield is obtained in the sample implanted with the maximum dose having an O:Er ratio equals to 9, as measured by secondary ion mass spectrometry.

In contrast, when the annealing is performed at 920 °C (Figure 3 right panel), the Er signal is almost independent with respect to the O dose and only the main peak at 1540 nm is clearly visible. Since the Er emission spectrum is strongly dependent on the environment, these measurements suggest that the higher annealing temperature might have induced the precipitation of non-optically active Er-O clusters.

### 4.2. Erbium Diode Electroluminescence

**Figure 4:**
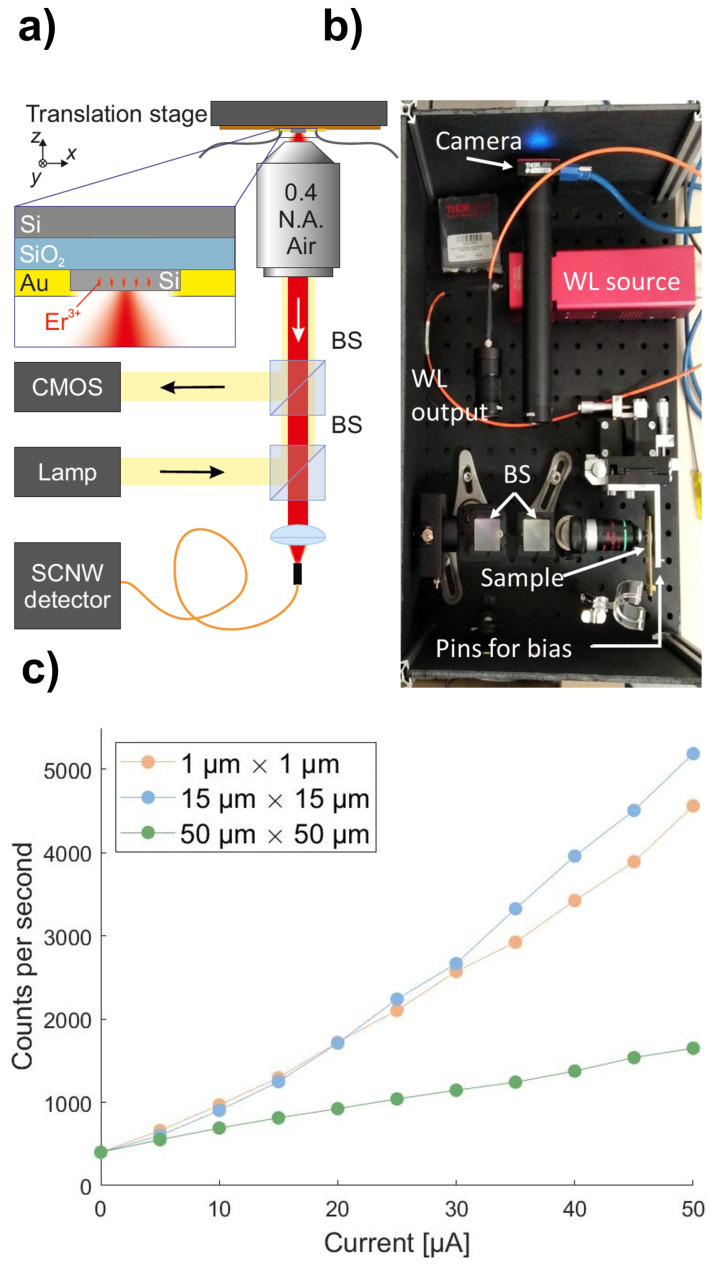
(**a**) Sketch of the experimental setup. (**b**) Picture of the the experimental setup (top view). (**c**) Counts collected per second in each device as a function of the current flux measured on the diode.

We have measured the emission from photodiodes implanted with a dose of 1 × $10^{13}$ Er cm${}^{-2}$ and 1.4 × $10^{14}$ O cm${}^{-2}$, annealed at 900 °C for 30 min. The implantation energies were 300 keV and 40 keV, respectively. The photodiodes are biased using a low noise wavefunction generator (Keithley sourcemeter 2450) operating in continuous wave, which also measures the flowing current. Each diode is biased to obtain a current from 0 to 50 µA (equivalent to a bias voltage from 0 V to about 1.2 V). To collect the emission, we employ a home-made optical microscope coupled to a superconducting nanowire single photon detector (SNSPD) through a standard single model fiber at 1550 nm (see Figure 4a,b). While the sample is maintained at room temperature, the SNSPD detector is cooled down to about 0.8 K to reach the superconducting state. In these operating conditions, the detector has a background noise of about 400 counts/s and has a quantum efficiency of about 95%. The emission is collected through an air objective with numerical aperture (NA) of 0.4 and imaged into the fiber core through an achromatic doublet with NA = 0.15, which matches the NA of the fiber, to minimize the coupling losses. To align the emission from the photodiode into the collection fiber, we first couple a 1550 nm fiber laser into the collection fiber and focus it on the sample to maximize the photoinduced current read by the wavefunction generator. The lateral alignment as well as the focus optimization is obtained using a manual translation stage (TAM-605SL—OPTOSIGMA Aluminum Crossed ROller XYZ stage) on which the sample is mounted. To measure the emission of the photodiodes we remove the beam-splitters (BS) employed for the sample alignment, to minimize the optical losses in the detection path.

As mentioned in Section 3, given the large step in refractive index between silicon (*n* = 3.4) and air (*n* = 1), most of the photons emitted by erbium centers remain trapped and guided within the silicon and therefore not emitted in free-space. For an optical interface such as this, the amount of photons emitted at a wavelength around 1550 nm in air is about 6% of the generated ones. In addition, the finite numerical aperture (NA = 0.4) of the objective allows for the collection of only 30% of these photons; therefore, only 2% of the emitted photons are collected by the objective. Given the transmittivity of the objective (30% at 1550 nm) and the coupling losses into the collection single mode fiber, we estimate an overall throughput (emitted photons/detected photons) of about 0.1%. We detect the emission from all the presented samples, with a lateral size of 1, 15 and 50 µm. The emissions in counts per second are shown in Figure 4c as a function of the current flowing in the device. Considering the magnification of our system (about 2.5:1) and the collection fiber core size (about 10 µm in diameter), we estimate that the region of the sample from which we collect photons has a diameter of about 4 µm.

By employing the translation stage, we could further optimize the signal, and, thanks to the small collection area, we verified that the maximum emission for the larger diodes (15 and 50 µm) occurs close to the electrodes in coincidence with the p-doped region, as expected. Since photons are emitted as a consequence of electron-hole recombination events in the depletion region, the total photoluminescence yield is expected to be proportional to the electric current density in the device or, at fixed current intensity, to its inverse cross section. This explains why a smaller signal is collected from the 50 µm × 50 µm device with respect to the 15 µm × 15 µm one, considering that the collection area of the optical setup is the same for both devices. Conversely, the 1 µm × 1 µm device is smaller than the objective collection area; therefore, one should expect a deviation with respect to the above-mentioned scaling law with the cross section. Nonetheless, this observation does not justify the observed number of counts per second, which is comparable with the 15 µm × 15 µm device and appears to be smaller with respect to the value one should expect. This behavior is still under investigation. Most probably, the fabrication process delivers better quality devices for larger areas and the realization of smaller devices might still require some optimization.

Considering the 1 µm device with an imposed current equals to 50 µA, taking into account that in the collection area (about 1.6 × $10^{-7}$ cm${}^{-2}$), we estimate the presence of about 10${}^{6}$ Er${}^{3+}$ ions. Since the emitted photons are about 5 × $10^{6}$, we evaluate that the number of the photons emitted per ion per second is about 5. We recall that, given the sample geometry, most of the emitted photons are expected to be lost in silicon contacts as demonstrated by the simulation performed by Lumerical. In addition, as reported in our previous work [14], at these specific co-doping levels only one in two erbium ions can be considered as optically active. Therefore, we conclude that a very conservative estimate of the number of photons emitted per active ion is about 10 per second. Considering the losses due to the finite numerical aperture of the objective and the photons lost in the contacts, we expect to reach a value compatible with our previous results at room temperature (1 × 10${}^{4}$ photons per second per ion) [14]. These values are still far from a typical QKD source that used short (<1 ns) and narrow bandwidth (<1 nm) laser pulses attenuated to the single photon level (µ ≃0.6) and with a repetition rate $\nu_{\mathrm{rep}}>$ 50 MHz, and further optimizations are surely required to reach acceptable levels. A first improvement would be to collect the emitted photons in an embedded waveguide. This could be a step forward towards a fully compatible CMOS light source for QKD applications where no demanding bit rates are required.

## 5. Conclusions

To conclude, we presented a silicon LED based on the electroluminescence of Er:O complexes operating at room temperature. The fabrication process is fully-compatible with CMOS technology and is performed on a 220 nm silicon-on-insulator wafer. The kinetic energy for ion implantation was designed in order to align the peak of the erbium doping profile with the center of the SOI. This result paves the way towards the integration of 1550 nm weak sources in silicon photonics for quantum communication applications.

## Data Availability

Data underlying the results presented in this paper are not publicly available at this time but may be obtained from the authors upon reasonable request.

## Abbreviations

The following abbreviations are used in this manuscript:

QKD	Quantum Key Distribution
SOI	Silicon On Insulator
LED	Light Emitting Diode
PL	PhotoLuminescence
SRIM	Stopping and Range of Ions in Matter
FDTD	Finite-Difference Time-Domain
NA	Numerical Aperture
BS	Beam Splitter
NA	Numerical Aperture
SPAD	Single-Photon Avalanche Diode
SNSPD	Superconductive Nanowire Single Photon Detector
